# IonPad adhesive gripper with variable-stiffness endoskeleton

**DOI:** 10.3389/frobt.2026.1815258

**Published:** 2026-07-21

**Authors:** Yuno Takitani, Keisuke Koyama, Weiwei Wan, Kensuke Harada

**Affiliations:** Department of Systems Innovation, Graduate School of Engineering Science, The University of Osaka, Toyonaka, Japan

**Keywords:** gripper, ionPad, robotic picking, temperature, variable stiffness material

## Abstract

In this study, we develop an innovative adhesion gripper capable of handling plate-like objects with various thicknesses without causing damage. The gripper combines an adhesion pad utilizing intermolecular forces and a variable-stiffness endoskeleton, allowing for precise adjustment of the pressing force during contact and the adhesion force while gripping an object. Central to its design is the fluid-driven and origami-structured endoskeleton that regulates contact and adhesion forces by controlling the fluid temperature. Furthermore, the gripper includes an outer frame which can be used for releasing the grasped objects by contracting the endoskeleton through fluid aspiration. Experimental evaluations demonstrate that the maximum contact force can be adjusted over a 27-fold range, while the adhesion force is tunable by up to a 12-fold variation. Notably, the ability to finely modulate the contact force facilitates the effective handling of thin plate components even on uneven surfaces. These results highlight the gripper’s potential for applications that require the delicate and precise manipulation of fragile, thin objects.

## 1 Introduction

Industrial products are typically composed of a wide variety of components, each with distinct physical properties such as shape, size, and material. In the context of robotic assembly, this diversity often necessitates the use of task-specific grippers tailored to individual components. However, as the number of components increases, so does the number of required grippers, significantly reducing the practicality and scalability of automated assembly, especially for products with a large number of parts.

This study addresses this limitation by focusing on the gripping and assembly of flat panels—components that are common in many products and exhibit considerable variation in thickness. We propose a versatile gripper mechanism capable of handling and assembling flat panels with a broad range of thicknesses using a single design. Unlike grippers with suction pads, which are unsuitable for such panels due to the risk of leaving suction marks, the proposed system employs an adhesion-based gripper that utilizes IonPad, a composite adhesive material composed of a functional resin coating and a base substrate, exhibiting adhesive properties driven by both chemical and physical interactions. However, to securely grasp thick panels, the gripper must press against the surface with a high contact force and a long contact duration to ensure sufficient adhesion before lifting. In contrast, thin panels are prone to damage when subjected to excessive force, and therefore must be handled with a lighter touch, requiring the gripper to apply minimal pressing force and lift them gently.

Considering these aspects, combining variable stiffness with adhesive gripping appears highly effective for the adaptive manipulation of panel-like objects with varying thicknesses. For realizing variable stiffness, phase-transition-based materials such as shape memory polymers (SMPs) can achieve continuous and reversible changes in stiffness with simple thermal control, making them suitable for compact systems. In addition, a gripper with SMP has the following additional merits. First, since it can realize very low stiffness, the gripper with SMP can press a thin panel with a very small pressing force. Second, they are advantageous in terms of miniaturization and fine stiffness modulation. In this study, we use a thin-sheet SMP (HUMOFIT®Mitsui Fine Chemicals Inc, 2024) combined with a water circulation system to accelerate thermal response and thereby improve the stiffness modulation speed of the actuator.

**FIGURE 1:**
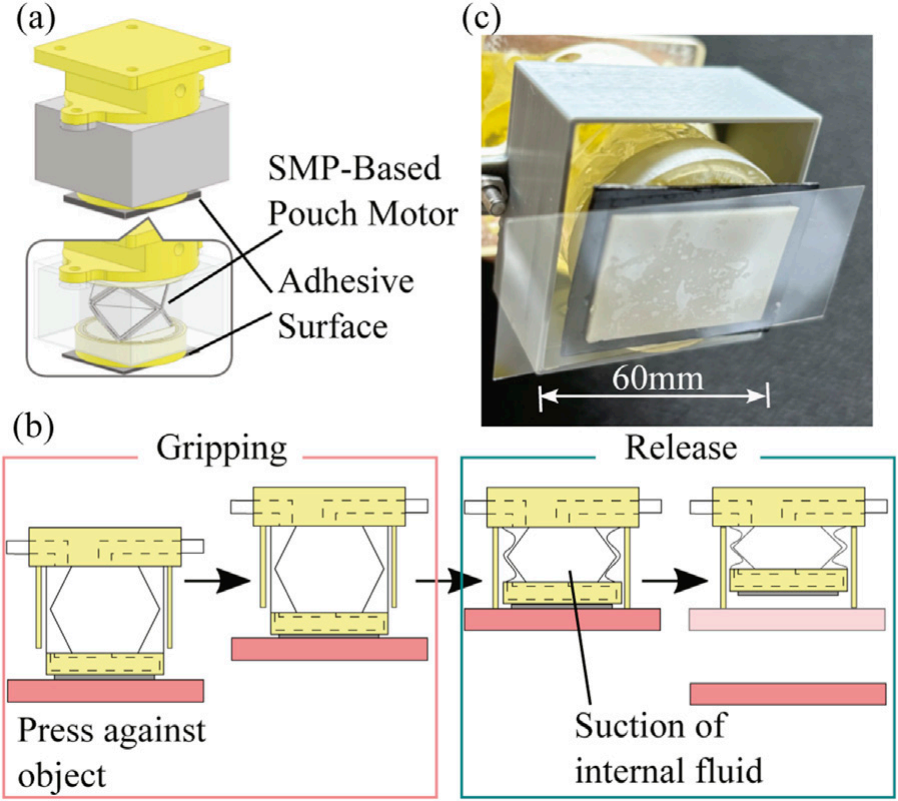
**(a)** Structure of the proposed gripper, highlighting the adhesive surface and the SMP-based pouch motor. **(b)** Schematic illustration of the gripping and release process, demonstrating the adhesion and detachment sequence using the adhesive surface. **(c)** Demonstration of the gripping for a flat object using the developed gripper prototype.

The proposed gripper employs an SMP-based pouch actuator to modulate stiffness and a surface-mounted IonPad to grip and release objects. The overview of the proposed gripper is shown in Figure 1. The actuator contracts upon fluid evacuation, enabling object detachment, while pressing the object against the adhesive surface initiates gripping. This sequence allows for programmable control over contact and release forces.

As demonstrated in Figure 1, the developed gripper can successfully grip flat objects through this combined mechanism. The key feature of the system lies in its ability to modulate contact force through thermal control of the SMP’s viscoelasticity. By adjusting the temperature of the working fluid, the actuator stiffness is varied, thereby altering the contact force applied to the object. Since the adhesive force of the IonPad depends on the contact force, this enables indirect control over adhesion. As a result, the system can reduce potential damage to delicate objects during release by weakening adhesion when necessary.

In this paper, we describe the design and fabrication of the proposed gripper. To evaluate its mechanical characteristics, a series of experiments was conducted. In one experiment, the actuator was pressed against a force gauge to measure how the contact force varied with actuator temperature and speed. In another experiment, the actuator equipped with an IonPad was pressed onto and detached from a flat surface to evaluate the adhesive force. The results demonstrate that the proposed system can dynamically regulate adhesive interaction through temperature-based stiffness control, offering a flexible and gentle gripping solution suitable for objects with varying fragility and shape.

## 2 Related work

Grippers based on suction and adhesion have been extensively studied as effective means for manipulating flat objects in industrial and research settings. This section outlines the related works in three primary axes: the development of suction and adhesion mechanisms (such as vacuum suction or gecko-inspired adhesion), the design of adaptive gripper bases that support compliant and robust contact, and the integration of variable-stiffness materials that enable controllable contact and adhesion forces.

### 2.1 Evolution of suction and adhesion techniques

Vacuum suction is one of the most mature and widely adopted adhesion methods, and has been implemented in numerous commercial products (Schmalz GmbH, 2024; PISCO Co., Ltd, 2024). Its ability to switch rapidly between gripping and releasing makes it suitable for high-speed applications. Recently, alternative suction mechanisms such as Bernoulli chucks (Davis et al., 2008) and closed-cup suction systems (Koivikko et al., 2021) have been developed to improve versatility and enable usage in various environments, including liquids.

Gecko-inspired dry adhesives have garnered significant attention due to their ability to function without external power sources and their compatibility with porous surfaces (Song et al., 2014; Glick et al., 2018). These microstructured surfaces exploit van der Waals forces to achieve strong yet reversible adhesion (Yu et al., 2011), and have been extended to multi-finger robotic hands in which adhesive patches provide both gripping and in-hand manipulation (Han et al., 2022). Recently, IonPad technology (Creative Technology Co., Ltd, 2024) has been introduced as a versatile adhesive material that responds to both contact force and duration, offering tunable adhesion in diverse environmental conditions. IonPad shares similarities with dry adhesion systems such as gecko-inspired structures in that it can achieve gripping without an external power source. However, unlike purely physical dry adhesion, IonPad also incorporates chemical interactions, allowing for adjustable adhesion strength based on contact conditions. These developments reflect a growing interest in adhesion methods that balance environmental adaptability, low-energy operation, and surface compatibility.

### 2.2 Variable-stiffness materials for adaptive gripping

A critical challenge in adhesion-based gripping is reconciling the conflicting requirements of high contact force (needed for strong adhesion to thick or heavy objects) and low contact force (needed to avoid damaging fragile thin objects). Phase-transition-based variable-stiffness materials have emerged as a promising solution to this challenge.

Shape memory polymers (SMPs) can achieve continuous and reversible changes in stiffness with simple thermal control, making them suitable for compact systems. Linghu et al. (2020) demonstrated a universal SMP gripper in which a heated, compliant polymer block conforms to and embeds objects of arbitrary shape; cooling then locks the grip, while reheating releases objects via shape recovery. Focusing specifically on the interplay between stiffness and adhesion, Coulson et al. (2022) presented a soft gripper whose thermoplastic composite contact pad is softened to maximize contact area before being cooled to generate high adhesive load capacity—demonstrating that thermally controlled stiffness can be directly leveraged to tune pull-off force. Mohammadi Nasab et al. (2023) further investigated an unstructured SMP membrane whose stiffness is modulated by a Peltier element, achieving a high-to-low adhesion ratio exceeding 2000 on curved substrates. Beyond polymers, (Liu et al. 2020) proposed a gripper with paraffin-filled variable-stiffness joints driven by shape-memory alloy wires, demonstrating an approximately 18-fold stiffness enhancement and confirming the broad applicability of phase-change composites for load-capacity improvement.

Despite these advances, existing SMP-based grippers typically require structural fingers or embed the target object within the bulk polymer, and do not address the specific challenge of handling flat panels with widely varying thicknesses using a single, compact surface-adhesion end-effector.

### 2.3 Pouch motors and origami-inspired actuators

Pouch motors, first proposed by Niiyama et al. (2014), are a family of fluidic soft actuators fabricated from heat-bonded sheet materials. Their flat, printable construction enables simple mass fabrication, and their force-displacement characteristics have been well characterized analytically. Li et al. (2017) generalized this architecture into fluid-driven origami-inspired artificial muscles, showing that embedding a compressible origami skeleton inside a sealed pouch skin enables contraction exceeding 90% of the initial length together with stresses of approximately 600 kPa—performance comparable to natural muscle. Subsequent work by Tokoro et al. (2023) integrated a variable-stiffness endoskeleton into a pouch motor, establishing the foundational actuator concept on which the present gripper is built.

Robertson et al. (2021) integrated origami modules with pneumatic pouch actuators to create modular reconfigurable robots, while Jiao et al. (2021) increased the contraction ratio and force output of pouch motors by incorporating bio-inspired origami folds into the longitudinal edges of the pouches.

The Yoshimura origami pattern—a tessellation of isosceles triangles that forms a cylindrical three-dimensional structure when wrapped circumferentially—has attracted particular interest as an actuator backbone because of its high axial compliance and strong torsional stiffness (Zhang et al., 2021). Zhang et al. (2023) proposed a rigid-soft hybrid origami actuator based on the Yoshimura pattern augmented with a parallel endoskeleton, achieving directional force output and thermally switchable stiffness. Luan et al. (2024) demonstrated that selectively activating and deactivating origami creases with variable-stiffness fibers enables a single actuator to switch among multiple morphing modes, further expanding the design space for stiffness-programmable soft actuators.

### 2.4 Limitations in gripper adaptability

While adhesion methods have diversified, many studies have assumed a relatively fixed configuration of the gripper base, often relying on rigid structures. This rigidity limits the ability of such grippers to adapt to object thickness or surface geometry. To address this, flexible or deformable gripper bases have been proposed (Seibel et al., 2020; Yoo et al., 2023), incorporating soft robotics elements such as pneumatic chambers and elastic structures. These designs enable some level of mechanical compliance, which can passively accommodate surface irregularities or height variations.

However, most of these flexible bases rely solely on passive deformation, with limited control over contact force or gripping stiffness. This restricts their ability to adjust gripping behavior depending on object properties—especially important when handling both delicate thin materials and heavier, thicker components within the same system. Furthermore, despite the promising characteristics of IonPad, existing implementations typically use rigid mounting, which diminishes their adaptability to varying object geometries.

### 2.5 Summary and research gap

Recent research has shown a clear trend toward enhancing the environmental and surface adaptability of adhesion-based grippers through the integration of variable-stiffness materials (Coulson et al., 2022; Linghu et al., 2020; Nasab et al., 2023), compliant actuator structures (Jiao et al., 2021; Robertson et al., 2021), and origami-inspired mechanics (Zhang et al., 2023; Luan et al., 2024; Zhang et al., 2021). Nevertheless, the challenge of handling flat objects with diverse thicknesses remains insufficiently addressed. Most existing approaches either fix the gripper base geometry or lack mechanisms for dynamically adjusting contact force. These limitations highlight the need for a new class of grippers that combines environmental versatility, controllable adhesion, and adaptive stiffness, particularly for the handling of delicate or variably-sized flat objects—which is the focus of the present work.

## 3 Proposed gripper

In this study, we propose an IonPad-based adhesive gripper that enables adjustment of both contact force and adhesion force through the modulation of the viscoelasticity of the gripper base. The base of the gripper consists of a pouch motor incorporating a variable-stiffness endoskeleton. A pouch motor is an actuator that achieves extension and contraction by pressurizing and depressurizing a sealed pouch, which encloses a foldable zigzag-shaped skeleton.

### 3.1 Gripper configuration

**FIGURE 2:**
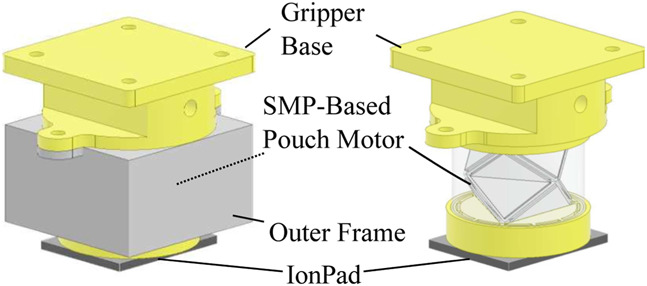
External appearance (left) and internal structure (right) of the gripper body.

The gripper consists of a variable-stiffness pouch motor, an adhesive surface, and an outer frame, as shown in Figure 2. The pouch motor with the IonPad is housed within the outer frame.

#### 3.1.1 Pouch motor configuration

**FIGURE 3:**
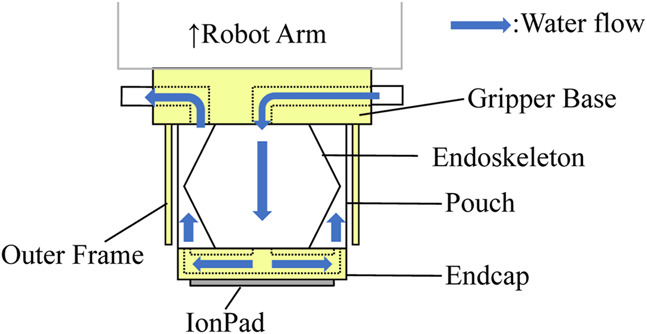
Configuration of gripper.

**FIGURE 4:**
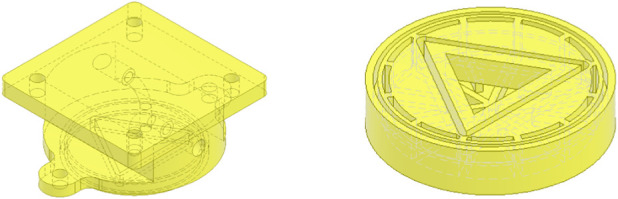
Gripper base (left) and endcap (right).

The detailed structure of the gripper is shown in Figure 3. The pouch motor comprises a cylindrical nylon-poly pouch (30 mm in diameter), a cylindrical origami-inspired endoskeleton, and end caps and gripper bases to secure both ends. Figure 4 show the appearance of the gripper base and end caps.

The gripper base includes two 2.5 mm diameter holes for water supply and two 4 mm diameter ports for tubing. Soft urethane tubes (outer diameter 4 mm, inner diameter 2.5 mm) are inserted and sealed with tape. One tube supplies the interior of the endoskeleton, while the other supplies the space between the skeleton and the pouch. The end cap is hollow, allowing water to circulate from the inner to the outer region of the skeleton, as illustrated in Figure 1. An IonPad (30 mm vertical, 20 mm horizontal) is affixed to the underside of the end cap using a slightly larger PVC plate.

#### 3.1.2 HUMOFIT®

The endoskeleton is made of HUMOFIT®, a thermosensitive shape memory polymer (Ferry, 1980). HUMOFIT®is a polyolefin-based sheet material having glass transition temperature ( $T_{g}\approx$ 28 °C). Below $T_{g}$ , segmental motion of the polymer chains is kinetically frozen, placing the material in a glassy state with high stiffness. Above $T_{g}$ , increased free volume activates chain-segment mobility, causing a sharp reduction in elastic modulus and a transition to the rubbery state. This temperature-dependent stiffness change is quantitatively described by the Williams—Landel—Ferry (WLF) equation, which predicts that small temperature increments near $T_{g}$ produce order-of-magnitude shifts in viscoelastic properties. Consequently, HUMOFIT®retains its shape at room temperature while rapidly softening upon contact with the body, conforming to complex anatomical geometries in a thermally controlled manner.

#### 3.1.3 Endoskeleton and material properties

HUMOFIT®is molded into a cylindrical origami form. The folding lines are designed to be thin, while the other regions are thicker, enabling proper deformation during actuation. Further details on the material properties of HUMOFIT®and the endoskeleton geometry are provided in the fabrication section.

#### 3.1.4 IonPad

IonPad is attached to the endoskeleton. IonPad adheres to flat surfaces such as glass through intermolecular forces—primarily van der Waals forces—generated at the interface between its specially coated resin surface and the workpiece, requiring no external energy source such as vacuum suction or electrical power (Israelachvili, 2011). Van der Waals forces are electrical attractive forces arising at the interface, and to activate them the two surfaces must be brought within approximately 3–5 Åof each other—a distance less than one hundred-thousandth the diameter of a human hair. The smooth mirror finish of the adhesive-type IonPad maximizes the real contact area with the workpiece, thereby generating sufficient adhesion in both the normal and lateral directions. Because the gripping mechanism relies on the intrinsic chemical and physical properties of the resin coating rather than on pressure differentials, IonPad functions effectively even under vacuum conditions and is well suited for handling thin, flat workpieces such as silicon wafers and display glass.

#### 3.1.5 Outer frame configuration

The outer frame is fabricated in a rectangular shape that avoids interference with the pouch motor and IonPad during operation. It is fixed to the base and is used for the release mechanism described in Sec. 3.3. Its height is set such that the IonPad is exposed when the pouch motor is extended, and the adhesive surface is retracted inside the frame when the pouch motor contracts.

### 3.2 Water control system

**FIGURE 5:**
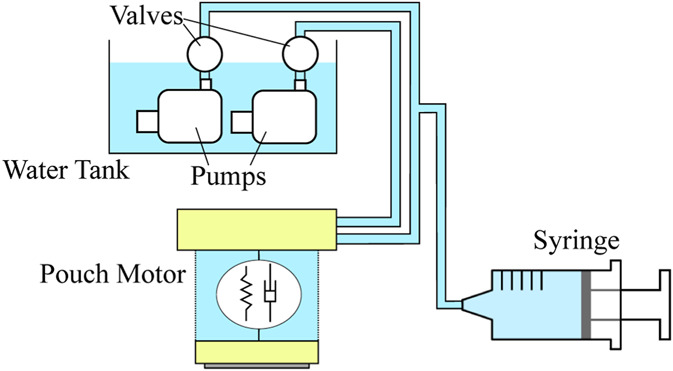
Water control system.

Water is used to control the expansion/contraction and temperature of the pouch motor. The water flow control system is illustrated in Figure 5. This system employs brushless pumps, solenoid valves, and a syringe actuated by a servo motor.

The system operates in three modes:(i) the flow channel is open but water is not circulated; (ii) the flow path is shut off; (iii) the flow channel is open and water is circulated.

Mode (ii) allows the pouch motor to contract via syringe operation, whereas mode (iii) is used to modulate contact force. Mode (i) is used during the gripping phase, while mode (ii) is applied during release.

A water tank is placed above the gripper so that the pouch motor can be filled by hydrostatic pressure. The syringe is driven by a servo motor (MX-64, Dynamixel), which is interfaced with a Windows PC via an Arduino Mega-compatible board for controlling displacement and rotation speed through serial communication.

In this work, we apply a pouch motor with a thermally tunable stiffness skeleton to an adhesive gripper. By integrating this actuator with a water control system that regulates both pressure and temperature, the gripper can perform gripping and release operations while simultaneously adjusting the contact force.

### 3.3 Operational modes

**FIGURE 6:**
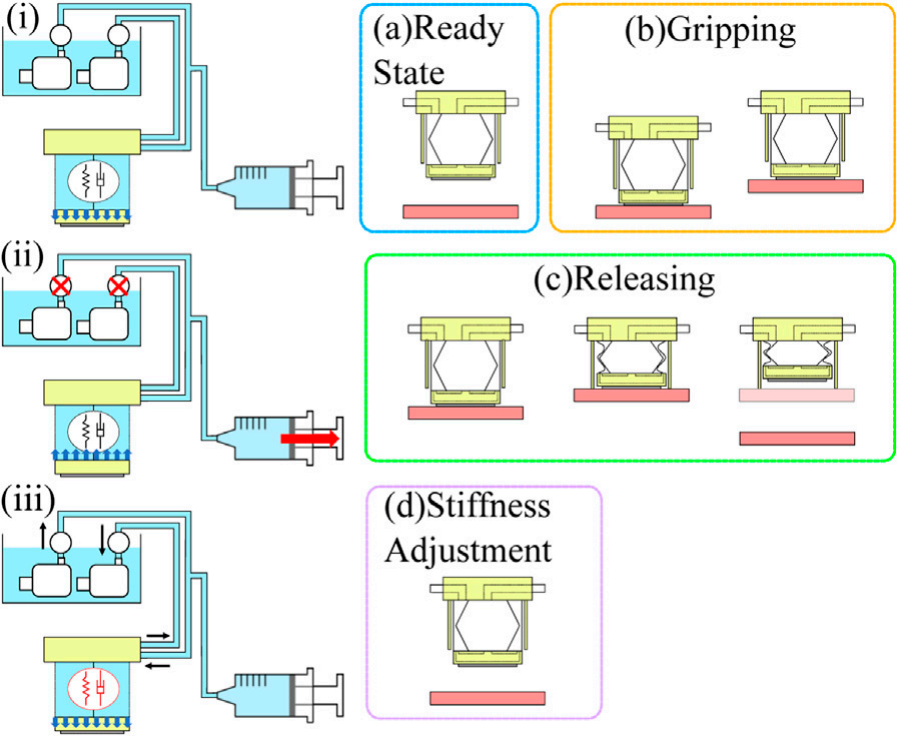
Gripper operating modes and corresponding water flow control. **(a)** Ready state, **(b)** Gripping, **(c)** Releasing, and **(d)** Stiffness adjustment. (i) Flow channel open without circulation, (ii) Flow channel closed with syringe operation, and (iii) Flow channel open with active water circulation.

The gripper adjusts its expansion/contraction and stiffness by controlling water flow and temperature. The relationship between the control system and gripper behavior is shown in Figure 6.

As shown in Figure 6, the gripper operates in four modes: (a) ready, (b) gripping, (c) releasing, and (d) stiffness adjustment by adjusting the fluid temperature. These modes allow the gripper to perform various tasks while controlling contact and adhesion forces.

#### 3.3.1 Operational mechanism

In the ready state, the water channel is open and the pouch motor passively extends due to hydrostatic pressure. Once fully extended, the adhesive surface is exposed outside the frame. The gripper is then positioned to bring the adhesive surface into contact with the target object.

During gripping, as the adhesive surface touches the object, the pouch motor deforms to absorb the impact. With the flow channel open, water can flow back, allowing the motor to contract. The contraction is limited to ensure the adhesive surface remains exposed beyond the frame, which depends on the frame’s length.

To release the object, the flow channel is closed and the pouch motor is actively contracted via syringe suction. The motor shortens, retracting the adhesive surface into the frame and detaching the object.

After release, the channel is reopened and the syringe returns to its initial position, restoring the gripper to the ready state.

### 3.4 Material and fabrication

#### 3.4.1 Material

To realize variable stiffness in the proposed actuator, this study employs HUMOFIT®, an SMP (shape memory polymer) developed by Mitsui Chemicals, Inc. This olefin-based material is characterized by excellent water resistance, low weight, and ease of processing, making it suitable for integration into soft robotic structures.

HUMOFIT®exhibits a glass transition temperature $\left(T_{g}\right)$ of approximately 28 °C, and its mechanical properties change markedly with temperature. Above $T_{g}$ , the material becomes soft and elastic, while below $T_{g}$ , it hardens significantly, exhibiting a higher modulus and tensile strength. These properties enable the material to deform under heat and pressure, fix the deformed shape upon cooling, and recover the original shape when reheated. Additionally, when heated above approximately 80 °C and deformed, it can be permanently reshaped after cooling.

HUMOFIT®is available in two forms: a foamed variant (HUMOFIT®F Type) containing internal bubbles, and a non-foamed variant (HUMOFIT®A Type) with similar thermomechanical properties. In this study, HUMOFIT®F Typesheets are pre-treated with heat to stabilize their dimensions, as heating causes the bubbles to change shape and may induce shrinkage.

### 3.5 Forming of endoskeleton

#### 3.5.1 Geometry and design parameters

**FIGURE 7:**
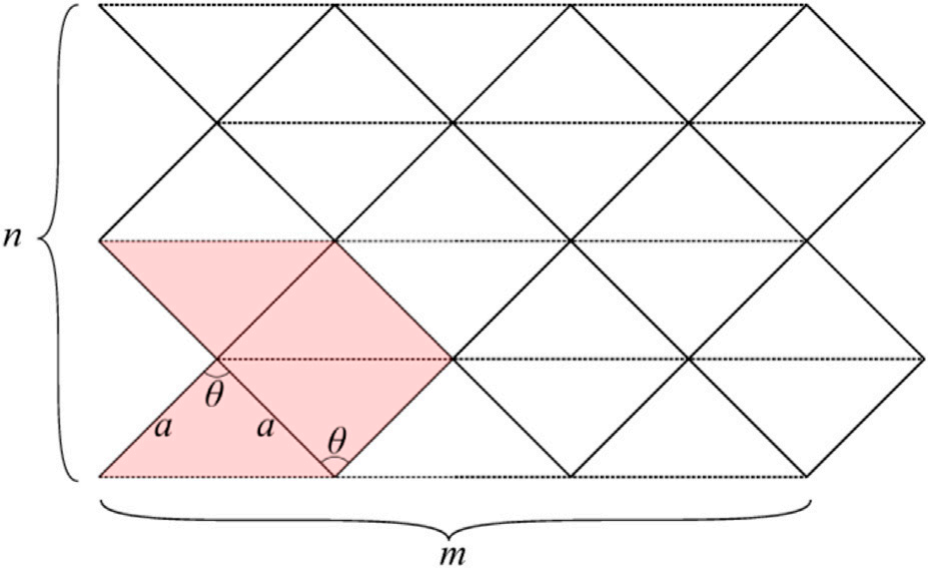
Development of yoshimura origami pattern. This pattern consists of repetitions of the basic pattern shown in red.

**FIGURE 8:**
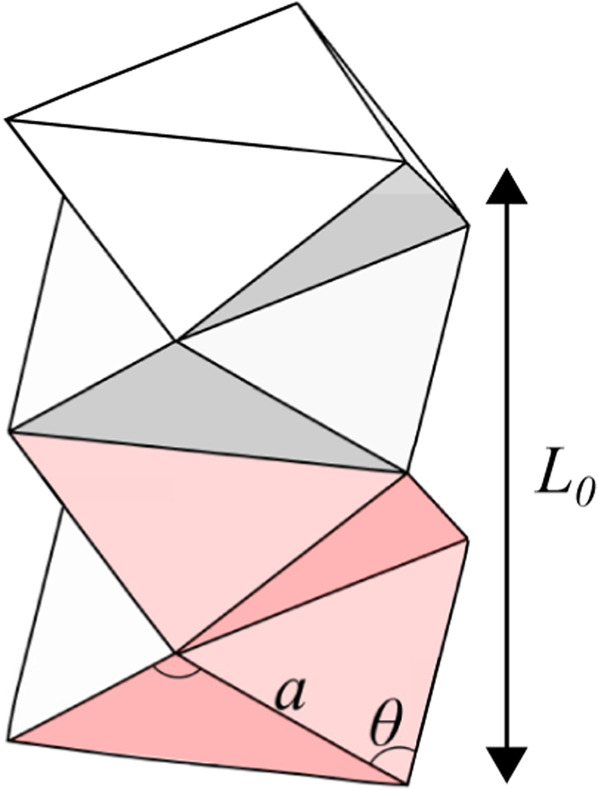
Three-dimensional view of yoshimura origami pattern. The area shown in red represents the basic pattern in Figure 7.

The endoskeleton is fabricated from bellows-shaped HUMOFIT®, designed to fold and unfold flexibly to provide mechanical compliance. Its geometry is based on a Yoshimura origami pattern, which consists of a tessellation of isosceles triangles arranged in a periodic, alternating fashion, as shown in the unfolded configuration in Figure 7. When this planar pattern is wrapped circumferentially, it forms a cylindrical three-dimensional structure, as illustrated in Figure 8.

To localize deformation within the folds, the structure is designed with thinner crease regions and thicker face regions. The creases are composed of HUMOFIT®A Type, while the face regions are reinforced by heat-welding HUMOFIT®F Typeon both sides of the HUMOFIT®A Type. A non-adhesive sandwiching method is used to prevent bonding between adjacent folded layers.

To ensure proper three-dimensional shaping and avoid interference between thickened faces, the crease width is adjusted relative to the fold thickness. Detailed design parameters used in this study are listed in Table 1, and the natural axial length of the structure is approximately 17.3 cm.

**TABLE 1:** Endoskeleton design parameters.

Number of axial patterns n	1
Number of circumferential patterns m	3
Isosceles length a	15 mm
Size of apex angle θ	90°
Width of fold line area d	0.5 mm
Thickness of HUMOFIT®F type $t_{\text{F}}$	0.4 mm
Thickness of $t_{\text{A}}$	0.4 mm

#### 3.5.2 Fabrication of origami pattern

**FIGURE 9:**
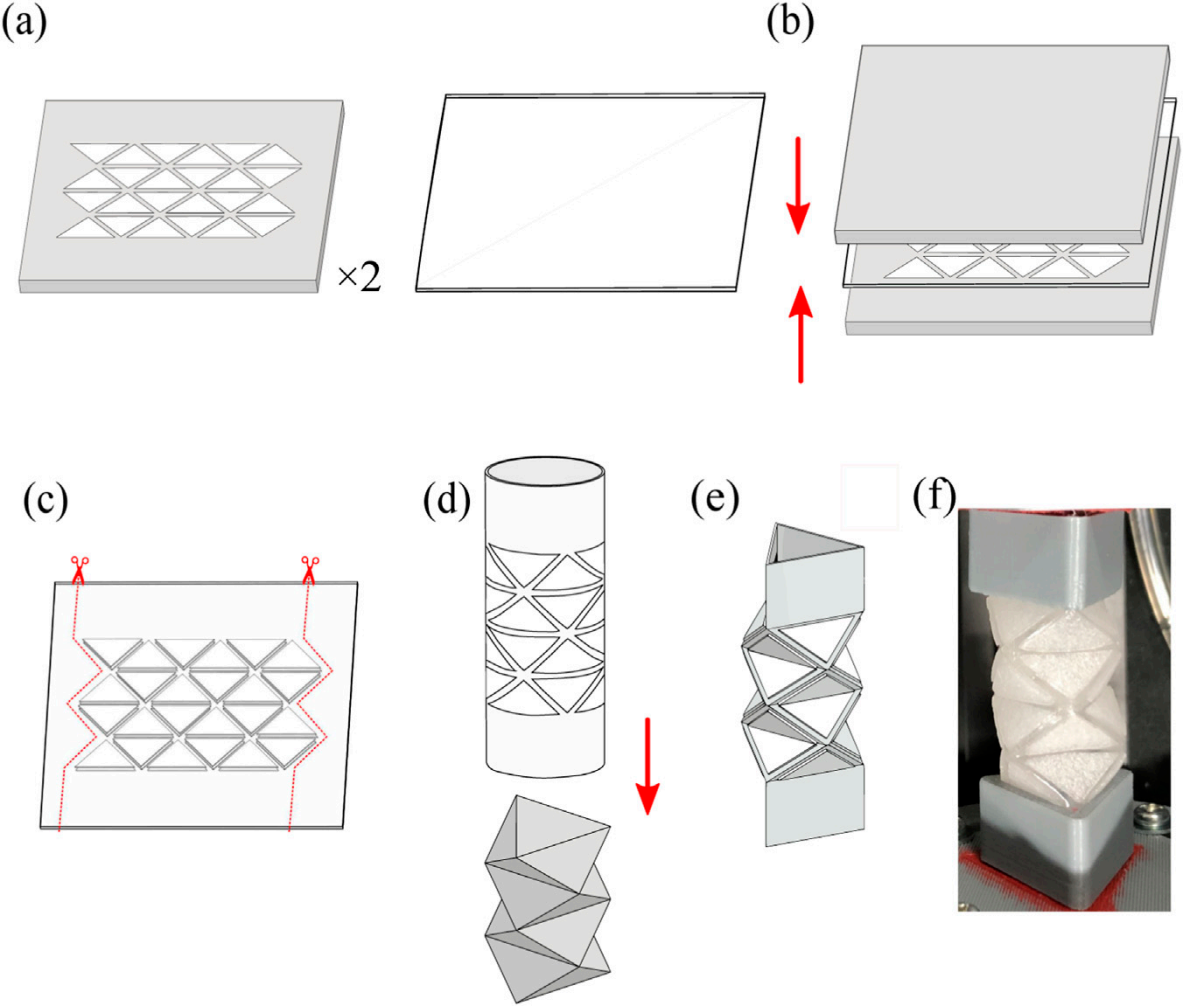
Fabrication procedure of the origami-patterned endoskeleton. **(a)** Cut HUMOFIT®F Typeand HUMOFIT®A Typesheets, and place the HUMOFIT®F Typesheets into the positioning mold. **(b)** Sandwich the HUMOFIT®A Typesheet between the HUMOFIT®F Type-lined molds and heat-weld the layers. **(c)** Remove the mold and trim the margins at both ends. **(d)** Heat-weld the ends to form a cylindrical shape, place the sheet into an origami mold, and heat it to memorize the shape. **(e)** After heating and natural cooling, remove the mold to complete the three-dimensional structure. **(f)** Picture of fabricated endoskeleton.

Because HUMOFIT®exhibits low flowability even at elevated temperatures, it is unsuitable for casting but suitable for reshaping by cutting, laminating, and thermal welding. In this study, the origami structure is fabricated through thermal bonding of sheets, followed by heat-forming using 3D-printed molds, as illustrated in Figure 9.

ABS-based molds (Z-ULTRAT, Zortrax) were 3D printed for positioning and forming. The positioning mold features concave Yoshimura patterns to align the HUMOFIT®F Typesheets. The HUMOFIT®F Typesheets (0.5 mm thick) were preheated at 90 °C for 10 min and allowed to cool naturally to prevent bubble deformation during subsequent processing.

The HUMOFIT®A Typesheet was cut to match the mold perimeter (Figure 9a), placed between two HUMOFIT®F Type-lined molds, and thermally bonded in an oven at 80 °C for 20 min (Figure 9b). The bonded sheet was then trimmed at both ends to a width of d – 2d (Figure 9c), heat-sealed, and placed in a cylindrical mold. The structure was reheated at 80 °C for 10 min and cooled naturally to memorize the 3D shape (Figures 9d,e).

#### 3.5.3 Gripper assembly

**FIGURE 10:**
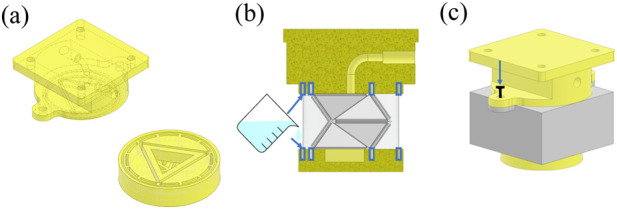
Assembly procedure of the gripper. **(a)** 3 days print the base and end caps. **(b)** Fix the endoskeleton and cylindrical film with epoxy resin. **(c)** 3 days print the outer frame and fix it to the gripper base.

The overall assembly process is shown in Figure 10. The gripper base and end caps were fabricated using a high-resolution 3D printer (Agilista, KEYENCE), which enables precise fabrication of parts with intricate internal features (Figure 10a). The endoskeleton and pouch were prepared separately.

The HUMOFIT®endoskeleton was manually actuated (folded and unfolded) 100 times to stabilize its mechanical response. It was then attached to the 3D-printed base and end caps. The pouch was made from nylon polyfilm, which was heat-sealed to form a cylinder of length $L_{0}+6$ mm. Grooves (0.6 mm wide and 3 mm deep) were prepared in the gripper base and caps to accommodate the film and skeleton ends.

These components were inserted into the grooves and fixed in place using an epoxy resin (Devcon ET, ITW Performance Polymers), which was allowed to fully cure, completing the assembly of the pouch motor (Figure 10b). Finally, the outer frame was printed using a 3D printer (M200 Plus, Zortrax S.A.) and affixed to the gripper base (Figure 10c).

## 4 Experiments

This section describes a series of experiments conducted to evaluate variations in contact force and adhesion force generated by the proposed gripper. Furthermore, the ability of the gripper to manipulate fragile objects, such as a cover glass, under non-uniform environmental conditions was also demonstrated by appropriately adjusting the contact force.

### 4.1 Preliminary experiments

Prior to the main experiments, preliminary investigations were performed to examine (i) the effect of repeated operation of the pouch motor and (ii) the influence of water tank placement on the contact force. These studies were conducted to ensure the reliability of the actuation system and to establish appropriate experimental conditions.

#### 4.1.1 Repetitive characteristics of the pouch motor

To evaluate changes in reaction force with repeated loading cycles, a repeated expansion and contraction tests of the pouch motor were conducted A force gauge (ZTA-200N, IMADA) was used to apply a vertical compressive force directly to the pouch motor without installing the adhesion pad and outer frame. The results indicate a decrease in contact force with an increasing number of cycles, particularly during the initial 10 trials. Based on these findings, a pouch motor preconditioned by 10 compression cycles.

#### 4.1.2 Placement of the water tank

In the pouch motor’s fluidic control system, the water tank is typically positioned at a higher elevation relative to the motor to prevent backflow when the flow channel is opened. This configuration introduces hydrostatic pressure at the base of the pouch motor, which in turn affects the contact force output during actuation.

To evaluate this effect, a simplified version of the pouch motor—excluding the internal endoskeleton—was tested using the same force measurement procedure described above. The height difference between the water tank and the actuator was varied among three conditions: 44cm, 22cm, and 0 cm. In addition, measurements were performed across a range of push-in speeds (1 mm/s to 9 mm/s) to examine the influence of fluid viscosity on actuation behavior.

**FIGURE 11:**
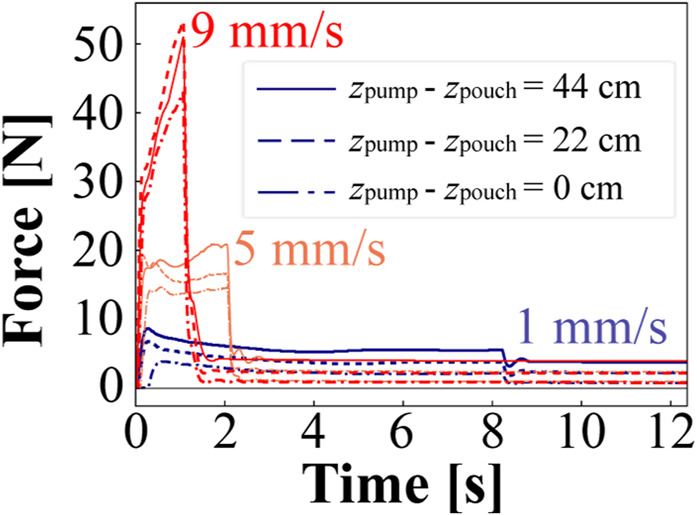
Time course of contact force on pouch without endoskeleton.

The temporal response of the contact force during compression is shown in Figure 11, where the vertical axis indicates contact force and the horizontal axis represents time elapsed from the start of compression. The results show that, due to the viscosity of the working fluid, contact force increased gradually during actuation and converged to a steady-state value after compression ceased. This steady-state force was found to be positively correlated with the height difference between the pouch motor and the tank. Additionally, higher compression speeds resulted in greater peak contact forces during actuation.

These results suggest that a smaller height difference allows for a lower minimum contact force and a broader range of contact force modulation. However, when the height difference was set to 0cm, the pouch motor failed to extend during the standby phase due to insufficient hydrostatic pressure.

Consequently, for all subsequent experiments, the height difference between the water tank and the pouch motor was fixed at 22cm, providing a balance between sufficient extension and adjustable contact force.

### 4.2 Method

#### 4.2.1 Contact force

**FIGURE 12:**
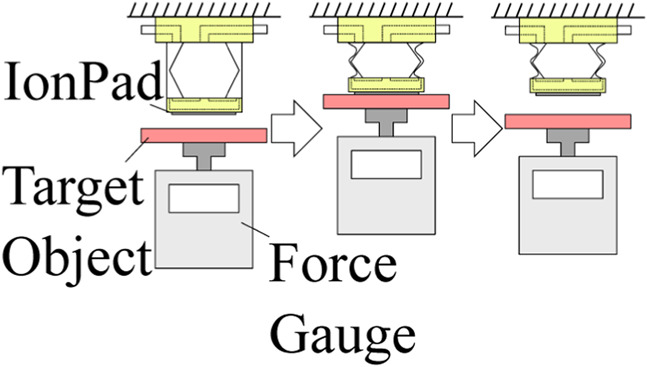
Flow of experiment on adhesion force.

As illustrated in Figure 12, the pouch motor—with its outer frame and adhesive surface removed—was fixed in a vertically downward orientation, consistent with the configuration used in the preliminary experiments. A compression force was applied from below using a force gauge (ZTA-200N, IMADA Co.), and the resulting contact force was measured. The displacement was set to 11mm, corresponding to the full stroke length of the pouch motor, and the push-in speed was maintained constant throughout the measurement.

#### 4.2.2 Adhesion force

The experimental setup shown in Figure 12 is also used. In addition, an IonPad was affixed to its lower surface of the pouch motor. A force gauge (ZTA-200N, IMADA Co.), with a vinyl chloride plate attached to its sensing tip (serving as the test object), was moved upward at a constant speed from a position 5 mm below the gripper until contact was established. After contact, the pouch motor was retracted at a constant speed.

During this sequence, both compressive and tensile forces were recorded, and the peak values of each were extracted. The maximum tensile force observed during detachment was used as the representative pull-off force. The contact duration was 0.5s, while the contact speed was varied between 1 mm/s and 9 mm/s. The detachment speed was set to 0.17 mm/s.

#### 4.2.3 Grasping a thin object

A cover glass (dimensions: 24 mm × 55mm, thickness: 0.12mm–0.17 mm) was placed on a bridge-shaped support. Grasping and releasing operations were performed using the proposed gripper. The internal water temperature of the gripper was controlled at either 35 °C or 25 °C, depending on the condition. The push-in duration was set to 1s, and the push-in speed was set to 9 mm/s. The height of the bridge section was 5 mm. After the push-in was completed, the solenoid valve and syringe were manually operated to perform the release.

Prior to each trial, the internal temperature of the pouch motor was adjusted by circulating heated or cooled water for approximately 1 minute. Although the temperature of the actuator body typically stabilized within about 30 s, a 1-min circulation period was employed to compensate for delays due to the length of the tubing and to ensure uniform thermal equilibration throughout the system.

### 4.3 Results

#### 4.3.1 Contact force

**FIGURE 13:**
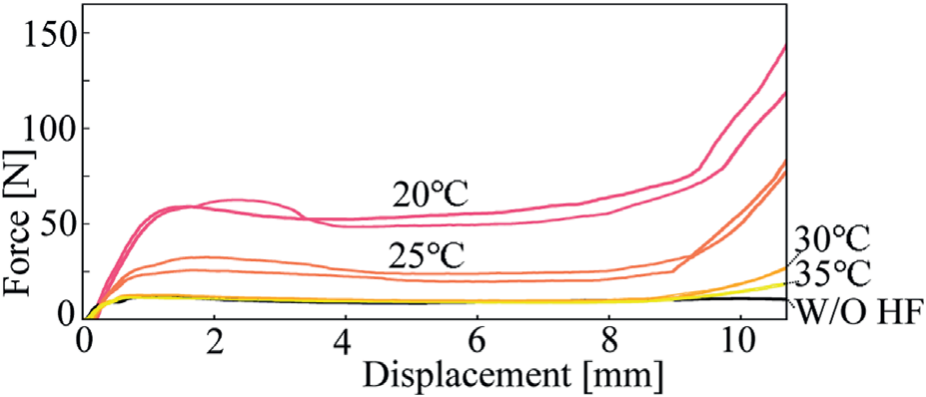
Compression force of pouch motor at constant push-in speed of 2.63 mm/s.

**FIGURE 14:**
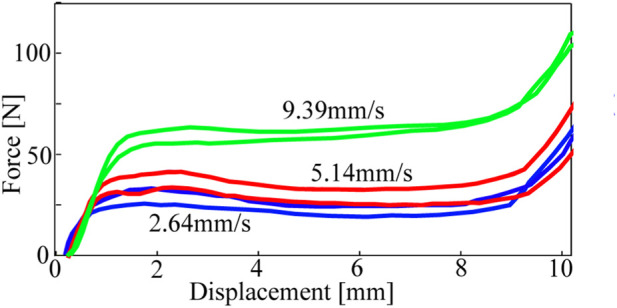
Compression force of pouch motor at constant temperature of 25 °C.

The contact force measured as a function of push-in displacement at a constant speed of 2.63 mm/s is shown in Figure 13. In this figure, the black curve represents the contact force measured from a pouch constructed without the HUMOFIT®endoskeleton. Figure 14 presents the temperature-dependent contact force for the pouch motor at a fixed temperature of 25 °C.

**FIGURE 15:**
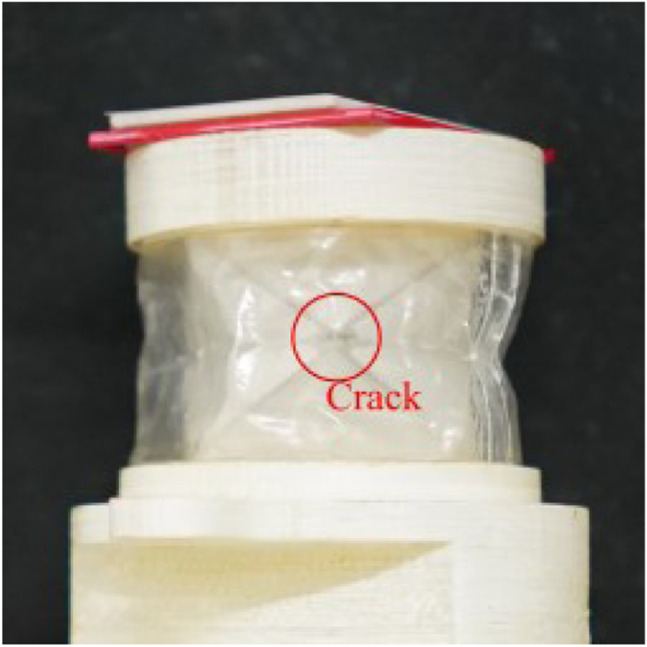
Broken pouchmotor.

From the results shown in Figure 13, it was confirmed that as the temperature decreases, the stiffness of the endoskeleton increases, resulting in a corresponding increase in contact force. In contrast, at temperatures exceeding 30 °C, the endoskeleton softens, and the measured contact force becomes nearly identical to the case without the endoskeleton.

In all conditions, the contact force increased with indentation depth as the endoskeleton approached its fully folded configuration. Additionally, as shown in Figure 14, higher indentation velocities resulted in increased contact forces, indicating a viscosity-dependent effect of the HUMOFIT®material even under isothermal conditions.

#### 4.3.2 Endoskeleton fatigue and failure

In addition to the reduction in contact force observed during repeated compression cycles, structural failure of the endoskeleton was also confirmed. When a thermoplastic polymer such as HUMOFIT®is subjected to repeated bending, cycle-by-cycle damage accumulates according to the linear damage summation rule (Palmgren–Miner rule) (Miner, 1945), and fracture occurs when the cumulative damage ratio reaches unity. At the microscale, repeated tensile stresses nucleate localized crazes—planar zones of fibrillated microvoids—which coalesce and propagate to form macroscopic cracks. Subsequent crack growth follows the Paris–Erdogan law $\left(da/dN=C\dot{\Delta }K^{m}\right)$ (Paris and Erdogan, 1963), and catastrophic fracture is triggered when the stress intensity factor range ΔK exceeds the material’s fracture toughness $K_{Ic}$ . Because HUMOFIT®operates near its glass transition temperature, viscoelastic hysteresis generates internal heat with each cycle; once the local temperature exceeds $T_{g}$ , markedly enhanced chain mobility accelerates crack propagation. This thermal effect is quantified by the WLF equation, which predicts that fatigue life at body temperature is substantially shorter than at ambient conditions. The damage typically began as a hole at the apex of the origami structure and propagated as cracks along the crease edges after about 300 cycles (Figure 15). This pattern of failure emerged after multiple actuation cycles, suggesting fatigue-induced degradation in the HUMOFIT®-based structure.

#### 4.3.3 Adhesion force

**FIGURE 16:**
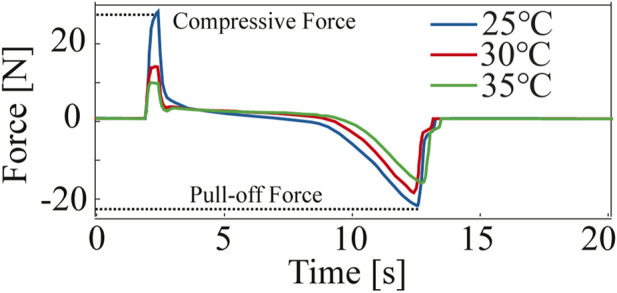
Time evolution of force gauge values at push-in speed of 3 mm/s.

The time evolution of the force measured by the force gauge during a grasping and detachment task involving a vinyl chloride plate is shown in Figure 16, with a constant indentation speed of 3 mm/s. The vertical axis indicates force, where positive values represent compressive force and negative values represent tensile force, and the horizontal axis denotes elapsed time. Each curve corresponds to a different temperature condition. During the indentation phase, the compressive force reached its peak, and during the detachment phase, the tensile force peaked.

**FIGURE 17:**
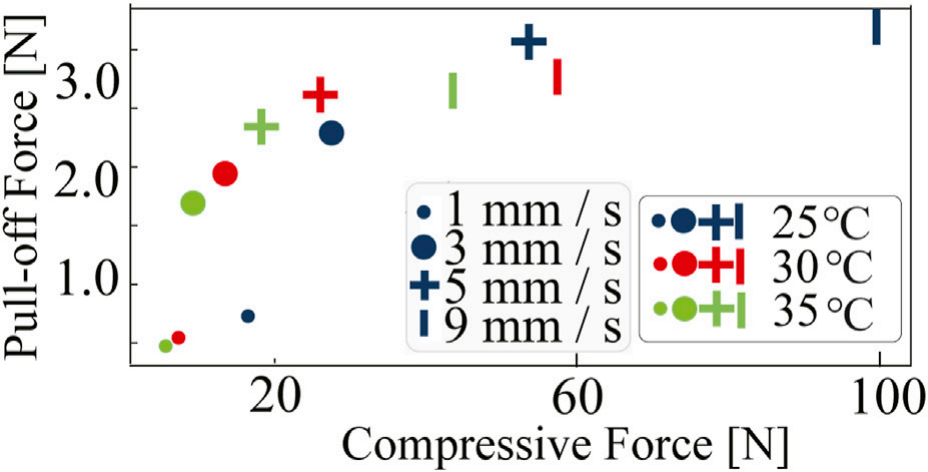
Relationship between maximum contact force and adhesion force on PVC board.

Figure 17 summarizes the maximum contact force and adhesion force obtained under various temperature and indentation speed conditions. The vertical axis represents adhesion force, while the horizontal axis shows the maximum contact force. Different symbols indicate different indentation speeds, and colors represent temperature conditions. The maximum contact force increased with increasing indentation speed and decreasing temperature. Correspondingly, the adhesion force also increased with increasing contact force. The maximum adhesion force recorded was 32.1 N at 25 °C and 9 mm/s, while the minimum was 4.3 N at 35 °C and 1 mm/s.

**FIGURE 18:**
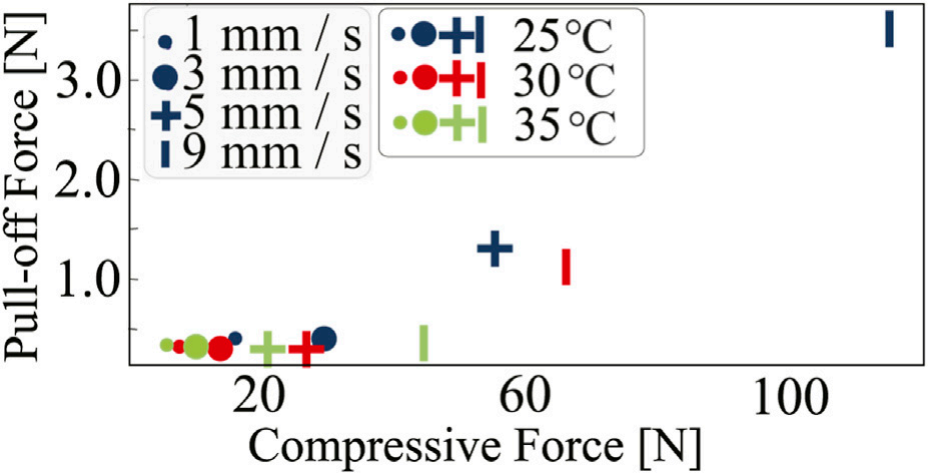
Relationship between maximum contact force and adhesion force on copy paper.

To see the object with a smaller adhesion force, Figure 18 shows the results when a sheet of copy paper was attached to the surface of the vinyl chloride plate. Similar trends were observed, with the maximum contact force increasing with higher indentation speed and lower temperature, and the adhesion force increasing accordingly. However, the absolute value of the adhesion force was reduced compared to the bare vinyl chloride case. The maximum adhesion force was 3.6 N at 25 °C and 9 mm/s, and the minimum was 0.3 N at 35 °C and 1 mm/s.

#### 4.3.4 Grasping a thin object

**FIGURE 19:**
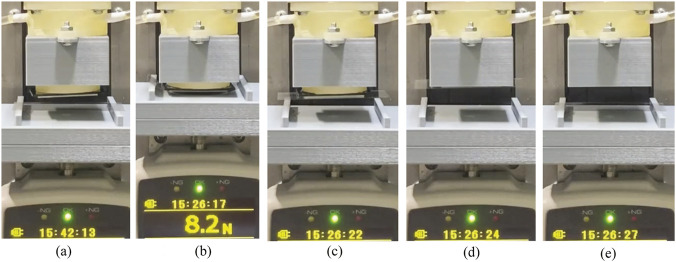
Successful case of grasping a cover glass with 35 ° C. **(a)** Initial state **(b)** Endoskeleton is pressed against the object **(c)** Object is lifted up **(d)** Outer frame contacts the object **(e)** Object is detached.

**FIGURE 20:**
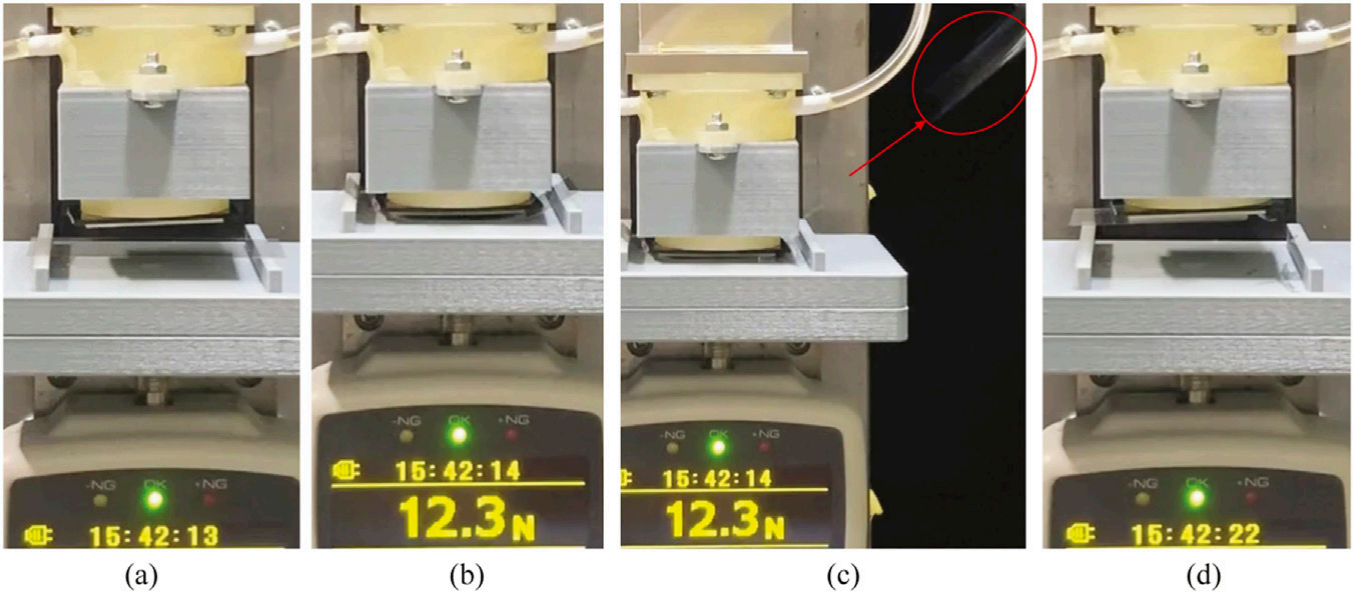
Failure case of grasping a cover glass with 25 ° C. **(a)** Initial state **(b)** Endoskeleton is pressed against the object **(c)** Object is broken and partially flies away **(d)** Broken object is lifted up.

We have performed the grasping experiment of a thin plate for twelve times with different conditions; water temperature, indentation speed and push-in duration. The results are shown in Table 2. The thin plate has to be carefully treated, and always failed to grasp when the water temperature is 25 °C. In addition, when the indentation speed is 3 mm/s, the grasping was failed for the water temperature 35 °C. Figures 19, 20 show the snapshot of two of the cases. At water temperature 35 °C, indentation speed 9 mm/s and push-in duration 1.0s, the gripper successfully grasped and released the cover glass without damage. In contrast, at water temperature 25 °C, indentation speed 9 mm/s and push-in duration 1.0s, the glass fractured upon contact.

**TABLE 2:** Picking experiment of cover glass; F1:picking was not successful, F2:releasing was not successful, and F3:glass was broken.

	3 mm/s	5 mm/s	9 mm/s
(a) Push-in duration 0.5s.			
35 °C	F1	✓	✓
25 °C	F1	F1	F2
(b) Push-in duration 1.0s.			
35 °C	F1	✓	✓
25 °C	F1	F2	F3

In both conditions, deformation of the cover glass was observed during the push-in phase. However, under the higher temperature condition, the pouch motor passively contracted upon contact, which helped reduce the applied impact and thereby prevented breakage. The maximum reaction force transmitted to the bridge-shaped base was 11.6 N at 35 °C, compared to 13.9 N at 25 °C.

## 5 Discussions

In repeated compression tests, the endoskeleton of the pouch motor exhibited structural failure, typically beginning with damage at the apex of the origami structure and spreading through the creases. This is likely due to stress concentration caused by the geometric design. Improving durability may be possible by modifying the folding pattern or adjusting design parameters to reduce localized deformation.

The increase in contact force with decreasing temperature and higher indentation speed is attributed to the temperature-sensitive viscoelasticity of HUMOFIT®. As the material becomes stiffer and more viscous under these conditions, it resists deformation more strongly. The adhesion force also increased with contact force, reflecting the IonPad’s force- and time-dependent adhesive behavior.

Experiments using a copy paper surface showed a notable reduction in adhesion force, suggesting that performance is sensitive to surface texture. Since the effective contact area contributes directly to adhesion, IonPad size is therefore considered a key geometric parameter that should be adjusted to match the surface characteristics of the target object.

Two operational issues were observed at low temperatures: slower contraction due to high viscosity, and insufficient retraction caused by air ingress. The former may be mitigated by dynamically controlling temperature—lowering it during contact for stiffness, and raising it for quicker release. Nevertheless, temperature changes are delayed by system constraints, which limits responsiveness. The latter issue could be resolved by improving sealing or maintaining higher temperatures, which prevented air contamination in the experiments.

When particles accumulate on the IonPad surface, the adhesion force decreases; the supplier therefore recommends periodic cleaning Creative Technology Co., Ltd., 2024. The stable special resin coating does not rely on adhesive layers, allowing repeated use; if particles adhere to the surface, the pad can be cleaned with pure water or industrial alcohol.

### 5.1 Contextualizing the contribution relative to existing systems

The contact force variation of 27-fold and the adhesion force variation of 12-fold, reported in this study, reflect the continuous tunability of the system through a single control variable—fluid temperature. We emphasize here that our contribution lies in what the lower bound of contact force enables, and in the passive mechanical behavior that emerges at elevated temperature.

Existing variable-stiffness grippers based on thermoplastic composites or SMP membranes have demonstrated effective stiffness and adhesion tuning for objects of moderate robustness (Coulson et al., 2022; Mohammadi Nasab et al., 2023). However, the challenge of handling thin, fragile flat panels—where the permissible contact force is extremely small—has not been directly addressed in those works. In the present study, the gripper successfully grasped and released a cover glass without fracture under conditions of elevated fluid temperature ( 35 ° C) and appropriate indentation speed (9 mm/s) , while the same gripper fractured an identical cover glass when operated at 25 ° C (Table 2; Figures 19, 20). This outcome is directly attributable to two mechanisms enabled by the variable-stiffness endoskeleton.

First, operating above the glass transition temperature reduces the stiffness of the endoskeleton, which lowers the contact force applied to the object. This demonstrates that the ability to reach a sufficiently low contact force, rather than the absolute range of tunability, is the functionally critical property for handling fragile objects.

Second, the softened endoskeleton above $T_{g}$ deforms passively upon contact with the object, absorbing the impact energy and preventing the transmitted force from exceeding the fracture threshold of the cover glass. Without our system, impact management during the approach phase relies on external motion control rather than on the mechanical properties of the gripper itself.

Taken together, these results suggest that the contribution of the proposed gripper is best characterized not by the magnitude of its force range, but by its ability to serve as a single end-effector that handles both fragile thin panels and thicker, heavier panels through thermal adjustment alone, without hardware reconfiguration. Thick panels require a high contact force and a long contact duration to build sufficient adhesion before lifting, while thin panels require a minimal contact force and passive impact absorption to avoid damage. The present system addresses both requirements within a unified design, which is the primary motivation stated in the Introduction and the aspect most difficult to replicate with grippers based on rigid mounting or passive compliance alone.

## 6 Conclusion

This paper proposed an adhesion gripper combining an IonPad with a pouch motor featuring temperature-dependent viscoelasticity. By attaching the IonPad to the tip of the motor, the gripper can adjust its contact and gripping forces based on changes in the stiffness of the internal structure. A water-based control system was used to drive the actuator and regulate temperature, enabling both motion and viscoelastic modulation.

Experimental results showed that contact and adhesion forces varied significantly with water temperature and indentation speed. The contact force changed by a factor of 27, and the adhesion force by a factor of 12, demonstrating the effectiveness of thermal and mechanical control in tuning gripping characteristics.

The gripper successfully handled a fragile cover glass without damage under suitable conditions, indicating its potential for manipulating delicate flat objects. Future work will investigate reproducibility across devices and optimize gripper geometry to improve durability and control precision.

## Data Availability

The raw data supporting the conclusions of this article will be made available by the authors, without undue reservation.
